# Supplementary material for: Novel conditional tabular generative adversarial network based image augmentation for railway track fault detection
Source: PeerJ Comput Sci. 2025 Jun 18;11:e2898. doi: 10.7717/peerj-cs.2898 (PMC12192886; doi:10.7717/peerj-cs.2898)
Supplement: Supplemental Information 3 [file peerj-cs-11-2898-s003.pdf]

Dear Editor,

We appreciate your time and consideration in reviewing our manuscript. We would like to formally request an authorship change by adding Dr. Basma as a co-author.

Dr. Basma has made significant contributions to the major revision of the manuscript. Specifically, she has conducted new experiments that have strengthened our study. Given her substantial intellectual and technical input, all authors unanimously agree that she deserves to be listed as a co-author.

New Author’s Contributions:

Conducted new experiments to enhance the study.

Provided critical insights and revisions to improve the manuscript.

Assisted in analyzing data and interpreting results.

Contributed to refining the discussion and conclusions.

We confirm that all authors, including Dr. Basma, have reviewed and approved the revised manuscript with the updated authorship.

Thank you for your consideration. Please let us know if any further information is required.

Best regards,

Dr. Ali Raza
